# Exploratory Impact of iCARE Nigeria, a Combined mHealth and Peer Navigation Intervention, on Depressive Symptoms and Substance Use Among Youth Living With HIV in Nigeria: Single-Arm Trial

**DOI:** 10.2196/71141

**Published:** 2025-07-29

**Authors:** Olusegun Ayomikun Ogunmola, Rita Frinue Tamambang, Kehinde Kuti, Lisa M Kuhns, Olutosin Awolude, Adedotun Adetunji, Bibilola Oladeji, Oladayo Olaleye, Adeola Mary Oyerinde, Robert Garofalo, Babafemi Taiwo, Olayinka Olusola Omigbodun

**Affiliations:** 1 Centre for Child and Adolescent Mental Health, College of Medicine, University of Ibadan, Ibadan, Nigeria; 2 Infectious Disease Institute, College of Medicine, University of Ibadan, Ibadan, Nigeria; 3 Department of Pediatrics, Feinberg School of Medicine, Northwestern University, Chicago, IL, United States; 4 Division of Adolescent Medicine, Ann and Robert H Lurie Children’s Hospital of Chicago, Chicago, IL, United States; 5 Department of Family Medicine, University College Hospital, Ibadan, Ibadan, Nigeria; 6 Department of Psychiatry, College of Medicine, University of Ibadan, University College Hospital premises, Queen Elizabeth II Road, Ibadan, 200212, Nigeria, 234 8132243158; 7 Division of Infectious Diseases, Feinberg School of Medicine, Northwestern University, Chicago, IL, United States

**Keywords:** mental health, depression, substance use, HIV/AIDS, youth, peer, cell phones, SMS, well-being, Nigeria

## Abstract

**Background:**

Mental health problems are a barrier to the well-being of youth living with HIV. Many youth living with HIV in Nigeria face peculiar biopsychosocial vulnerabilities that predispose them to mental health problems including depression and substance use. In addition to improving treatment outcomes like medication adherence and linkage to care, peer engagement has shown some promise in improving the social and emotional well-being of this population. Mobile health (mHealth) interventions like SMS text messaging medication reminders may also contribute to better outcomes in youth living with HIV. Emerging evidence suggests that combination interventions may be more effective than single interventions in improving key HIV testing and treatment outcomes among youth in Nigeria.

**Objective:**

This study aims to explore the impact of Intensive Combination Approach to Rollback the Epidemic in Nigerian Adolescents (iCARE Nigeria) study—an mHealth and peer navigation intervention primarily aimed at medication adherence and viral suppression—on depressive symptoms and substance use among youth living with HIV in Nigeria.

**Methods:**

A single-arm clinical trial was conducted at the Infectious Disease Institute, College of Medicine, University of Ibadan, Nigeria— primarily to improve medication adherence and viral suppression among youth living with HIV attending its HIV clinic. The intervention combined peer navigation and daily, 2-way, text message medication reminders delivered over a period of 48 weeks. Participants were screened at baseline and follow-up visits (24 and 48 weeks) for depression and substance use using standardized measures. Paired *t* tests and McNemar tests were used to investigate the change in depressive symptoms and the change in the proportion of participants reporting substance use over time, respectively.

**Results:**

All 40 enrolled participants (n=20, 50% male; mean age 19.9 y, SD 2.5 y) completed baseline and follow-up visits at week 24, while 37 (92.5%) participants completed the week 48 visit. Compared with baseline, there were significantly fewer self-reported depressive symptoms observed at 48 weeks (mean 2.89 vs 2.08; *t*_36_=2.04, 95% CI 0.006‐1.615) but not at 24 weeks (mean 2.89 vs 2.62; *t*_36_=0.47, 95% CI –0.74 to 1.44). There were fewer self-reports of substance use at weeks 24 and 48 when compared to baseline, but these were not statistically significant (odds ratio [OR] ∞, 95% CI 0.189-∞ and OR 3.0, 95% CI 0.24‐157.49, respectively).

**Conclusions:**

These findings suggest a statistically significant reduction in depressive symptoms among youth living with HIV over the 48-week intervention period that may be due to the iCARE Nigeria intervention. However, given limitations such as low levels of depressive symptoms at baseline, small sample size, and the lack of a control group, future studies such as the randomized stepped wedge evaluation of the iCARE intervention are needed to provide better insights into these exploratory findings.

## Introduction

Mental health problems are common among youth living with HIV attributed to a combination of biological and psychosocial factors [1]. Biologically, HIV infection may have a direct impact on the brain, resulting in behavioral disturbances, mood disorders, neurocognitive decline, and psychosis [2]. In addition, some side effects of antiretroviral therapy (ART) used to achieve viral suppression in HIV infection may have mental health implications for youth living with HIV [3]. Psychosocial factors, which may contribute to the heightened risk of mental health problems among youth living with HIV, include high rates of orphanhood (and its attendant lack of social and material needs), overburdened caregivers, stigma and discrimination, and disclosure of HIV status [4-7].

Studies conducted in sub-Saharan Africa have reported prevalence rates of depression or probable depression among youth living with HIV ranging from 4.4% to 30.2% [1,8-12] compared to 5.8% to 20% among seronegative youth [11,12]. Substance use has also been found to co-exist with mental health problems in youth living with HIV [13], although this relationship is understudied among youth living with HIV in sub-Saharan Africa. Mental health problems (including depression and substance use) have been linked with poor medication adherence, higher risk sexual behaviors as well as reduced quality of life among youth living with HIV, leading to loss to follow-up and even premature mortality [14]. Despite well-documented evidence of mental health problems faced by youth living with HIV, access to mental health services remains an often-unmet priority need for youth living with HIV [15].

There is growing evidence showing that a combination of peer engagement and mobile health (mHealth) interventions may be useful in improving emotional and psychosocial well-being of youth living with HIV [16]. A recent study piloted a peer-led mHealth psychosocial intervention for youth living with HIV aged 15‐20 years in South Africa, demonstrating increased emotional support and a safe and nonjudgmental atmosphere that encouraged discussion of intimate topics like disclosure and adherence [16]. Peer engagement has also been found to positively impact psychological well-being in addition to improving linkage to care, medication adherence, viral suppression, and retention in care among youth living with HIV [14,17]. Therefore, informed with evidence that combination interventions may be more effective than single interventions in improving key HIV testing and treatment outcomes among youth in Nigeria [18,19], the Intensive Combination Approach to Rollback the HIV Epidemic in Nigerian Adolescents (iCARE Nigeria) study developed, locally adapted, and piloted for efficacy 2 youth-specific interventions—to increase HIV testing among youth at risk of HIV infection and to promote viral suppression in youth living with HIV— each with mHealth and peer navigation components [20,21].

Therefore, in this paper, we explore whether the iCARE medication adherence and viral suppression intervention may have secondary beneficial effects on the mental health of youth living with HIV, specifically by reducing depressive symptoms and substance use among the participants.

## Methods

### Study Design

The methodology for the iCARE Nigeria pilot study has been described in detail in earlier publications [20,21]. One of the iCARE Nigeria interventions was a single-arm trial, designed to test the efficacy of a combination of peer navigation and daily SMS text messaging medication reminders in improving medication adherence and viral suppression (defined as plasma HIV-RNA of less than 200 copies/ml) among the youth living with HIV [20]. Participants completed 3 study visits—baseline, 24 weeks, and 48 weeks. Data were collected between June 2019 and August 2020 using an interviewer-administered survey, deployed using Research Electronic Data Capture (REDCap; Vanderbilt University) [22,23].

### Study Site

This study was conducted at the Infectious Disease Institute, College of Medicine, University of Ibadan (IDI-CoMUI), which receives support from the Centers for Disease Control (CDC)-funded AIDS Prevention Initiative in Nigeria (APIN) Public Health Initiatives and provides ART to more than 6000 persons with HIV, aged 15 years or older.

### Study Participants

Participants were youth living with HIV (15‐24 y old) who had been on ART for at least 3 months. Participants were selected via systematic random sampling. First, a sampling frame containing a list of eligible youth living with HIV at IDI-CoMUI was created from the medical records, which was then stratified by status—virally suppressed versus viremic. Subsequently, every “nth” potential participant was then selected based on the final ratio of target sample size (n=40) to eligible patients in the sampling frame. After a number of refusals and no-shows, the target sample size of 40 with only 35% (14/40) of the participants being virally suppressed (ie, viral load <200 copies/mL). Being a pilot study, the sample size was primarily chosen, not to have adequate power to establish statistical significance but based on predetermined effect sizes satisfactory for future full-blown investigation as indicated in the analysis of the primary outcome (viral suppression) at the study endpoints [20].

### Intervention

A combination of peer navigation and daily, 2-way, SMS text messaging medication reminders was delivered over a period of 48 weeks. The daily SMS text messaging reminders were personalized, coded, and scheduled based on the time the participants were expected to take their medication. A total of 15 minutes after receipt of the personalized reminder, a second personalized message was sent asking the participants if they had taken their medication and each participant was expected to provide a reply. An encouraging message tailored to the response then followed. Furthermore, 8 peer navigators (each assigned 5 peers) were trained to provide peer support, based on a personalized needs assessment and action plan, including facilitating referrals to services as necessary.

The peer navigators undertook a formal training that included topics such as: using coping skills; identifying signs of common mental health problems (depression, anxiety, alcohol and substance use disorders, and suicidality); and providing peer support to youth living with HIV who may be showing signs of mental health problems. Peer navigators checked in on their peers at least once a week, typically over the phone, but in-person meetings could also happen depending on the needs and peculiarities of the peer. Peer navigators were supervised and supported by the designated members of the study team who met with them monthly. At baseline and then again at 24 weeks, study coordinators administered a needs assessment questionnaire, which sought to identify the needs of each participant across social, educational, physical health, and mental health domains. To determine whether a participant would benefit from mental health support, the form asked if the participant in the past two weeks had (1) little interest or pleasure in doing things and (2) was feeling down, depressed, or hopeless. The questionnaire also asked if participants had used alcohol or other psychoactive substances within the previous 6 months. An action plan was then developed and used to prioritize each participant’s needs and goals and lay out a plan for achieving those goals. Study coordinators referred participants to mental health services, as needed.

### Ethical Considerations

The study was granted ethical approval by the joint University of Ibadan/University College Hospital, Ibadan, Ethics Committee (approval UI/EC/21/0050) and Northwestern University, Lurie Children’s Hospital (approval 2019‐2446). All participants provided informed consent, consistent with the 2014 Nigeria Federal Ministry of Health Guidelines [24]; minors (aged 16‐17 y or 15 y and emancipated) provided consent for themselves without parental involvement. All participants were assured of their right to withdraw from the study at any given time without the risk of any consequences. All study data were deidentified (each participant received a unique study ID separate from their clinic ID) and stored on the REDCap database accessible only by authorized study staff. At each study visit, participants received light refreshments and payments to cover their transportation costs.

### Outcomes and Outcome Measures

#### Substance Use

The World Health Organization (WHO) Alcohol, Smoking, and Substance Involvement Screening Test (ASSIST) [25] was used to assess substance use. This tool measures use of 10 substances (ie, tobacco, alcohol, cannabis, cocaine, amphetamine, inhalants, sedatives, hallucinogens, opioids, and other) over the previous 3 months. For this analysis, the item, “In the past three months, how often have you used the substances you mentioned?” was used as a measure of substance use. A response of “never” was coded as “no substance use,” while those who selected any of the responses “once or twice,” “monthly,” “weekly,” “daily or almost daily” were categorized as endorsing recent substance use (yes or no). Responses to questions about all 10 substances were aggregated to indicate recent use of versus no recent use of substances. We also explored the types of substances used by the participants and how these changed over time.

#### Depressive Symptoms

The 10-item Center for Epidemiological Studies-Depression scale short version (CES-D) tool [26] was used to assess for depressive symptoms. The tool asks how the individual has felt or behaved within the past 1 week and has items which are scored on a 4-point Likert scale; that is, “rarely or none of the time (less than a day),” “some or a little of the time (1‐2 d),” “occasionally or a moderate amount of time (3‐4 d),” and “all of the time (5‐7 d).” For this analysis, the total score (range 0‐30) was used as a continuous measure, with higher scores indicating greater depressive symptoms and a score of ≥10 indicating probable depression as this has been found to be a valid cut-off in sub-Saharan Africa including among vulnerable youth [26].

### Data Analysis

Frequencies, percentages, and mean (SD) were used to describe participants’ personal characteristics. McNemar test was used to test for the changes in proportion of participants reporting substance use between baseline and the 2 follow-up periods (24 weeks and 48 weeks). Paired sample *t* tests were used to determine the changes in depressive symptoms between baseline and the 2 follow-up periods. Participants who died during the trial or were lost to follow-up were not included in the respective analyses. SPSS (version 21; IBM) was used for descriptive and paired *t* tests analyses, while R Studio (Posit) was used for the McNemar exact test computations—to generate CIs and odds ratios. All tests were conducted with significance set at .05 alpha level.

## Results

### Personal Characteristics of Participants

The study sample consisted of 40 youth living with HIV evenly distributed between male and female sex at birth. The mean age of the participants was 19.9 (SD 2.5) years. The majority (24/40, 60%) were students in senior secondary school (equivalent to grade 10 to 12, high school), more than half (22/40, 55%) were perinatally infected with HIV and 35% (14/40) were virally suppressed at baseline.

### Mental Health Profile of Participants

Upon the exclusion of 3 participants (1 dead and 2 lost to follow-up) in the final analyses, results showed that 2.7% (1/37) of the sample screened positive for probable depression (ie, had a score of ≥10 on the CES-D) at both baseline and 48 weeks study visits while no participant met the threshold for depression at 24 weeks. At baseline, 10.8% (4/37) of the participants reported they had used substances within the past 3 months, compared to 5.4% (2/37) who reported using alcohol and opioids at the 24-week visit and 2.7% (1/37) who reported using alcohol at the 48-week visit.

### Effect of the Intervention on Depressive Symptoms Among Youth Living With HIV

**Figure 1.**
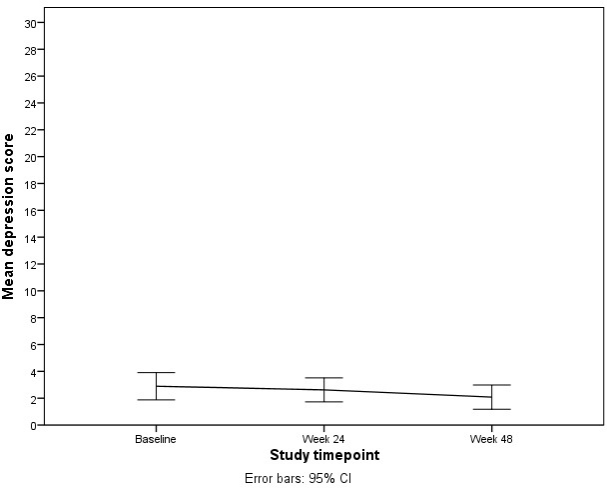
Changes in depression symptoms over time among study participants.

Table 1 shows the results of paired *t* test analyses for difference in depressive symptoms. Depressive symptoms reduced over the course of the intervention from a mean of 2.89 (SD 3.05) at baseline to a mean of 2.62 (SD 2.69) at 24 weeks (*P*=.64) and a mean of 2.08 (SD 2.71) at 48 weeks follow-up (*P*=.048). See Table 1 and Figure 1.

**Table 1.** Paired sample *t* tests showing the effect of the intervention on depressive symptoms (at 24 weeks and 48 weeks; n=37^a^).

Study timepoint	Depressive symptoms, mean (SD)	*t* test^b^ (*df*)	95% CI
Baseline	2.89 (3.05)	—^c^	—
24 weeks	2.62 (2.69)	0.47 (36)	−0.74 to 1.44
48 weeks	2.08 (2.71)	2.04 (36)	0.006 to 1.615^d^

a n<40 due to the exclusion of 3 participants (1 dead and 2 lost to follow-up).

b 2-tailed.

c Not applicable.

d Significant at *P*<.05.

### Effect of the Intervention on Substance Use Among Youth Living with HIV

At both week 24 and week 48 visits, there was no significant difference in the number of participants that endorsed the use of substances, but overall substance use was infrequently reported by participants at all assessments (see Table 2).

**Table 2.** McNemar exact test showing the effect of the intervention on substance use at 24 weeks and 48 weeks follow-up (n=37^a^).

	Substance use at week 24			Substance use at week 48		
	No, n (%)	Yes, n (%)	OR^b^ (95% CI)	No, n (%)	Yes, n (%)	OR (95% CI)
Substance use at baseline			∞ (0.189-∞)			3.0 (0.24‐157.49)
No	33 (100)	0 (0)		32 (97)	1 (3)	
Yes	2 (50)	2 (50)		3 (75)	1 (25)	

a n<40 due to the exclusion of 3 participants (1 dead and 2 lost to follow-up).

b OR: odds ratio.

## Discussion

### Principal Findings

Findings from the iCARE Nigeria pilot study demonstrated the initial efficacy of the iCARE treatment intervention in improving viral suppression among youth living with HIV in Nigeria [20]. In this analysis, we found a modest but statistically significant signal that the iCARE intervention may also have secondary beneficial effects on depressive symptoms. This finding is of potential significance because it was detected in a cohort of only 37 youth, and on average, the youth living with HIV reported minimal depressive symptoms at baseline.

The low (2.7%) prevalence of clinically significant depressive symptoms found in this study at baseline is remarkable when compared to the findings among youth living with HIV in many other sub-Saharan Africa populations. For example, studies have found rates such as 18.9% (Malawi), 27% (Tanzania), 6%‐11% (Cameroon), and 30.2% (Ethiopia) [9,11,12,27]. However, most of these studies were conducted among larger samples (ranging from 169 to 331 youth living with HIV), which afforded them greater statistical power, while some included only young women [1,8], unlike our study that included an equal proportion of men and women. Similar to our results, however, some studies in South Africa found 4%‐6% of adolescents screening positive for depression [1,8]. The low prevalence of depression found in those studies was partly attributed to the support youth living with HIV had been receiving in their respective HIV clinics, which many had been attending since early childhood [8]. A similar mechanism may underlie the low prevalence of clinically significant depression in our cohort. Specifically, the site of our study (IDI-CoMUI) has a youth club supporting adolescents with HIV for the past 6 years. In addition, mental health diagnoses (including depression) and long distance to health care facilities are some factors that have been associated with poor adherence to ART and retention in HIV care [28,29]. It is therefore possible that most youth living with HIV with these underlying disadvantages (and who were also more likely to have depression) were less likely to agree to participate in our study. Would the level of depressive symptoms be greater among youth living with HIV who are not as engaged in care or who receive care in a setting with less support? And would the iCARE intervention have had a greater impact on depressive symptoms among such youth living with HIV? These are important questions for future research to explore, including the ongoing randomized evaluation of the iCARE intervention, which is taking place at multiple sites in Nigeria including in settings that are not as advantaged as the current study site. Also, it may be important to note that 1 participant who had clinically significant depressive symptoms at baseline did not meet the cut-off for depression at week 24.

Furthermore, peer navigators were at the center of support provided by the iCARE intervention. The peer navigators were trained, among other things, to identify signs of common mental health problems, support peers to adopt healthy coping skills, provide emotional support to their peers, and facilitate referral to mental health services. The peer navigators may have contributed to the improvement in their peers’ mental well-being by deploying these competencies. This should be explored more fully in future research. The improvement may also be attributable in part to the mental health screening that was conducted as part of a needs assessment done at baseline and at the week 24 study visit. Notably, 8 participants were referred for mental health counseling at baseline, including 7 who flagged positive on at least one of the depression screening questions on the needs assessment form. The eighth participant was referred for mental health counseling based on concerns raised during the needs assessment that her alcohol use might affect adherence to ART.

We also explored trends in substance use, although at baseline only 11% of participants reported substance use in the previous 3 months. Whereas studies conducted in South Africa have found a substance use disorder prevalence of 1.7%‐3% among adolescents living with HIV in South Africa [1,8], none of our participants met the cutoff for substance use disorder. Also, there was no significant change from baseline in the use of substances among participants at either week 24 or week 48. The fact that the tool to assess for substance use was interviewer-administered may have increased the likelihood of social desirability, thus resulting in low endorsement of substance use by the participants. However, given the peculiarities of the ASSIST screening tool, it had to be self-administered. We, however, tried to minimize social desirability by conducting interviews in a private setting—by study staff who had cultivated a largely trusting, safe, and nonjudgmental relationship with the participants over the course of the study. However, we acknowledge that in spite of these measures, social desirability bias (and consequently underreporting) could still have been present. While the reduction in substance use was not statistically significant, it may still have clinical and practical relevance [30]. This is supported by findings from our needs assessment and action plan, which reveal that most of the participants using substances (3 out of 4 at baseline and 1 out of 2 at week 24) expressed a priority goal to stop using substances. Considering the threat psychoactive substances pose to the health outcomes of youth living with HIV (such as through drug interactions and weakening of the immune system) [31], being able to get 3 out of 4 youth living with HIV to successfully achieve their goals of staying off alcohol and other such substances may be clinically meaningful.

### Limitations

Our findings are tempered by some limitations. First, this study is constrained by limitations of the study design (absence of randomization and a control group) and as such, the potential effect of confounding variables such as relatively good mental health at baseline (evidenced by the low level of reported depressive symptoms) and peer support received outside of the intervention could not be accounted for. In addition, the use of an interviewer-administered tool could have introduced some bias leading to underreporting of substance use and depressive symptoms. Similarly, while some mental health improvements were observed, the lack of an objective measure (for example, clinician-assessed depression) is another drawback. Furthermore, the fact that some participants did not complete all the assessments may have further skewed and reduced the generalizability of our findings. Finally, given our small sample size and the fact that there was only 1 participant with clinically significant depression at baseline, the clinical significance of our findings on depressive symptoms is uncertain and worthy of further exploration.

### Conclusion

This study found that the iCARE intervention, which combines peer navigation and SMS text messaging medication reminders, may improve depressive symptoms, and thus may have secondary mental health benefits among youth living with HIV in Nigeria. We saw no statistically significant impact on substance use among the participants in this small exploratory study. Given that positive mental health is a continuum that goes beyond the absence of psychopathology, any significant move toward flourishing and well-being [32] in this sample of vulnerable youth suggests potential mental health benefits that warrant further study. The ongoing, randomized, stepped-wedge study (N=568), which is comprehensively evaluating the iCARE Nigeria treatment intervention (NCT 04950153), would potentially provide further evidence for the efficacy of this intervention in promoting the mental well-being of youth living with HIV.

## Abbreviations

ART: antiretroviral therapy
ASSIST: Alcohol, Smoking, and Substance Involvement Screening Test
CES-D: Center for Epidemiological Studies-Depression scale short version
iCARE: Intensive Combination Approach to Rollback the Epidemic in Nigerian Adolescents
IDI-CoMUI: Infectious Disease Institute, College of Medicine, University of Ibadan
mHealth: mobile health
OR: odds ratio
REDCap: Research Electronic Data Capture
WHO: World Health Organization
